# Crystal Structure and Thermoelectric Properties of Novel Quaternary Cu_2_MHf_3_S_8_ (M—Mn, Fe, Co, and Ni) Thiospinels with Low Thermal Conductivity

**DOI:** 10.1021/acs.chemmater.1c03593

**Published:** 2022-02-15

**Authors:** Oleksandr Cherniushok, Oleksandr V. Smitiukh, Janusz Tobola, Rafal Knura, Oleg V. Marchuk, Taras Parashchuk, Krzysztof T. Wojciechowski

**Affiliations:** † Thermoelectric Research Laboratory, Department of Inorganic Chemistry, Faculty of Materials Science and Ceramics, AGH University of Science and Technology, Mickiewicza Ave. 30, Krakow 30-059, Poland; ‡ Department of Chemistry and Technology, Volyn National University, Voli Ave 13, Lutsk 43025, Ukraine; § Faculty of Physics and Applied Computer Science, AGH University of Science and Technology, Mickiewicza Ave. 30, Krakow 30-059, Poland; ∥ Department of Science, Graduate School of Science and Technology, Kumamoto University, 2 Chome-39-1 Kurokami, Chuo Ward, Kumamoto 860-8555, Japan

## Abstract

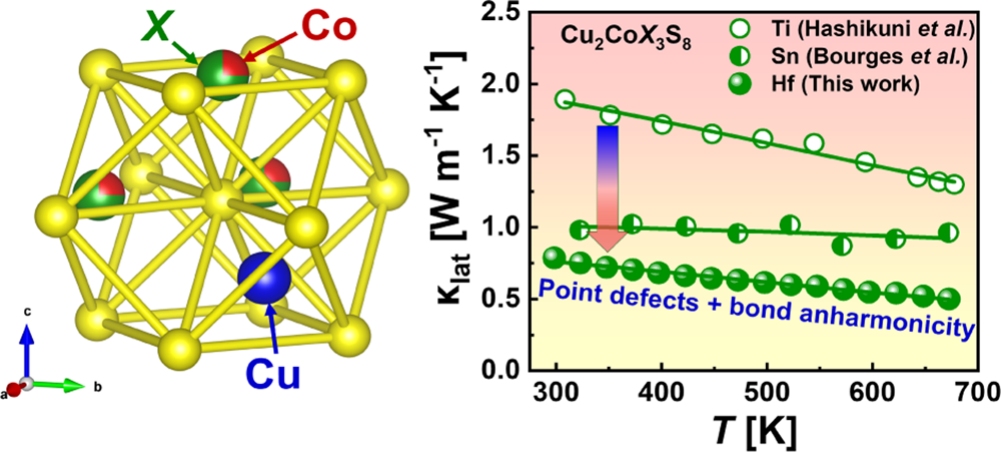

Uncovering of the origin of intrinsically low thermal conductivity in novel crystalline solids is among the main streams in modern thermoelectricity. Because of their earth-abundant nature and environmentally friendly content, Cu-based thiospinels are attractive functional semiconductors, including thermoelectric (TE) materials. Herein, we report the crystal structure, as well as electronic and TE properties of four new Cu_2_MHf_3_S_8_ (M—Mn, Fe, Co, and Ni) thiospinels. The performed density functional theory calculations predicted the decrease of the band gap and transition from p- to n-type conductivity in the Mn–Fe–Co–Ni series, which was confirmed experimentally. The best TE performance in this work was observed for the Cu_2_NiHf_3_S_8_ thiospinel due to its highest power factor and low thermal conductivity. Moreover, all the discovered compounds possess very low lattice thermal conductivity κ_lat_ over the investigated temperature range. The κ_lat_ for Cu_2_CoHf_3_S_8_ has been found to be as low as 0.8 W m^–1^ K^–1^ at 298 K and 0.5 W m^–1^ K^–1^ at 673 K, which are significantly lower values compared to the other Cu-based thiospinels reported up to date. The strongly disturbed phonon transport of the investigated alloys mainly comes from the peculiar crystal structure where the large cubic unit cells contain many vacant octahedral voids. As it was evaluated from the Callaway approach and confirmed by the speed of sound measurements, such a crystal structure promotes the increase in lattice anharmonicity, which is the main reason for the low κ_lat_. This work provides a guideline for the engineering of thermal transport in thiospinels and offers the discovered Cu_2_MHf_3_S_8_ (M—Mn, Fe, Co, and Ni) compounds, as new promising functional materials with low lattice thermal conductivity.

## 1 Introduction

The unique ability to convert heat into electrical power makes thermoelectric (TE) materials very promising for improving energy utilization and management.^1,2^ The efficiency of this compelling technology is determined by the TE materials’ figure of merit, *ZT* = σ*S*^2^*T*/(κ_lat_ + κ_el_), where σ is the electrical conductivity, *S* is the Seebeck coefficient, κ_lat_ and κ_el_ are the lattice and electronic components of the thermal conductivity, respectively, and *T* is the absolute temperature.^3^ To provide a high *ZT* parameter, the simultaneous enhancement of the power factor (*PF* = *S*^2^σ) and low lattice thermal conductivity κ_lat_ is necessary.^4−6^ The difficulty of producing high-performance TE materials is that the parameters σ, *S*, and κ_el_ are interdependent through the carrier concentration *n*. Moreover, the toxic nature of most well-established TE materials, that is, Bi_2_Te_3_, PbTe, and GeTe, and the drastic cost increase of tellurium in the last years, restrict their widespread utilization. Therefore, the search for new earth-abundant and Te-free TE materials is a great challenge.

Up to date, many efforts were applied for the development of materials that consist of earth-abundant and environmentally friendly elements. Following this idea, a lot of attention was focused on the development of sulfides, especially the copper-based sulfide compounds such as Cu_2–*x*_S,^7^ ternary Cu–Sn–S semiconductors,^8,9^ chalcopyrites,^10^ cubanites,^11^ colusites,^12^ stannoidites,^13^ tetrahedrites,^14^ argyrodites,^15,16^ and some other Cu-based sulfides.^17−20^ Despite good TE performance, some of these compounds (e.g., Cu_2–*x*_S and argyrodites) are superionic conductors and their practical application is restricted due to low thermal stability accompanied by cation migration, which causes the structure degradation of the material.^21,22^

The finding of more stable sulfides leads to ternary and quaternary transition metal thiospinels^23,24^ as the derivatives of the spinel MgAl_2_O_4_ structure type.^25^ Thiospinel compounds with the general formula AB_2_S_4_ can accommodate variable metal elements, which significantly tunes physical properties and demonstrates the playground for the designing of promising materials with high functionality.^26^ As an example, ternary Cu-based CuM_2_S_4_ (M—Ti, Cr, Co, etc.) thiospinels and their derivatives have gained significant interest due to their diversity of magnetic,^27,28^ catalytic,^29,30^ electrical,^28^ and TE properties.^19,31−35^ Moreover, the density ρ of thiospinels usually does not exceed ∼5.0 g/cm^3^, which is significantly lower compared to the other well-established TE materials, that is, Bi_2_Te_3_ (7.7 g/cm^3^),^36^ PbTe (8.24 g/cm^3^),^37^ GeTe (6.18 g/cm^3^),^38^ CoSb_3_ (7.6 g/cm^3^),^39^ and half-Heuslers (8.0–11.0 g/cm^3^).^40^ The use of low-density materials for the construction of TE modules can decrease the overall weight of devices. Only one trap that may occur here is that the low mass of elements usually corresponds with the high thermal conductivity of materials.^41^

Because of the aforementioned reasons, many studies related to the investigation of the crystal and TE properties of ternary and quaternary thiospinels have occurred recently. Wyzga et al. performed a systematic study on indium-based ternary thiospinels to explore their potential for TE applications. The high resistivity of the studied MIn_2_S_4_ (M—Mn, Fe, Co, and Ni) thiospinels together with quite high κ_lat_ (∼2.5–3.5 W m^–1^ K^–1^ at 298 K) lead to a low *ZT* < 0.1 at 760 K.^42,43^ Only In_0.67–0.33_In_2_S_4_ thiospinel alloyed with selenium showed improvement in the power factor and together with relatively low κ_lat_ (∼1.1–1.4 W m^–1^ K^–1^ at 298 K) resulted in a higher *ZT*_max_ = 0.25 at 760 K.^44−46^ However, Chen et al. reported for In_2.67–*x*_Cu_*x*_S_4_*ZT*_max_ = 0.5 at 700 K due to the increased power factor and suppressed κ_lat_ (∼1.1–1.4 W m^–1^ K^–1^ at 298 K).^47^ Hashikuni et al. recently reported *n*-type Cu_2_MTi_3_S_8_ (M—Mn, Fe, Co, and Ni) quaternary thiospinels as a TE material with a large power factor of 0.6 mW m^–1^ K^–2^. However, the relatively high κ_lat_ (1.4–2.3 W m^–1^ K^–1^ at 298 K) also results in a low *ZT* = 0.2 at 650 K for Cu_2_CoTi_3_S_8_.^31,48,49^

The performed analysis indicates that the first step in the optimization of the TE performance of the thiospinel materials and the other TE materials is connected with the intrinsically low thermal conductivity.^50^ The thermal conductivity of semiconductors is usually dominated by lattice thermal conductivity κ_lat_.^51,52^ Thus, the effective method for finding new, advanced TE materials is to search for semiconductors with low lattice thermal conductivity κ_lat_.^24^

The reported Cu_2_CoTi_3_S_8_ thiospinel compound crystallizes in the space group *Fd*3̅*m* (no 227, Pearson symbol *cF*56) and is characterized by a large number of atoms (*N* = 56 per cubic cell with *a* ∼ 10 Å) distributed over three crystallographic sites, that is, one tetrahedral site for Cu (8*b*), a mixed octahedral site for Ti/Co (16*c*), and one site for S (32*e*). The (CuS_4_) tetrahedra are corner shared with four [(Co/Ti)S_6_] octahedra, which share their edges. According to Spitzer,^41^ the relatively high coordination number of the M atoms in such a structure may favor low lattice thermal conductivity. Aiming to reduce lattice thermal conductivity, Bourges et al. substituted Ti for Sn, and showed that Cu_2_CoSn_3_S_8_ possesses lower κ_lat_ ∼ 1.0 W m^–1^ K^–1^^34^ compared with ∼1.9 W m^–1^ K^–1^ at 298 K for Cu_2_CoTi_3_S_8_.^31^ According to the radii of the Ti atom and the size factor of the octahedral form, the site that is occupied by Ti(Sn) atoms can be replaced by Hf atoms. Because of the higher atomic mass, a more effective reduction of lattice thermal conductivity than in the cases of Ti and Sn is expected.

The purpose of this study was to find environmentally friendly and mechanically stable materials with high earth abundance characterized by low lattice thermal conductivity, as this is the major request for high TE performance. Keeping this in mind, we successfully synthesized and characterized four new thiospinels with the chemical composition Cu_2_MHf_3_S_8_ (M—Mn, Fe, Co, and Ni). The structure of these compounds is based on the three-layer close packing of sulfur atoms. The nature of filling 1/2 octahedral voids with the statistical mixture of atoms *L* = Hf + M (M—Mn, Fe, Co, and Ni) and 1/8 tetrahedral voids with Cu, respectively, causes the differentiation of the unit cell in the structure into octants and the formation of smaller F-cubes. The large cubic unit cells full of vacant octahedral voids have been found extremely useful for the reduction of the lattice thermal conductivity through increased lattice anharmonicity. As a result, the thermal conductivity for the Cu_2_MHf_3_S_8_ (M—Mn, Fe, Co, and Ni) compounds shows significantly lower values compared to the other Cu-based thiospinels reported up to date.

## 2 Experimental Details

### 2.1 Materials and Synthesis

Samples with the nominal compositions of Cu_2_MHf_3_S_8_ (M—Mn, Fe, Co, and Ni) were prepared by melting high-purity Cu (shot, 99.99%), Mn (shot, 99.99%), Fe (shot, 99.99%), Co (shot, 99.99%), Ni (shot, 99.99%), Hf (shot, 99.99%), and S (shot, 99.99%) in quartz containers evacuated to a residual pressure of 10^–2^ Pa. The total mass of every sample was 3 g. The ampules with the stoichiometric mixtures of elements were heated up to 1423 K at the rate of 12 K/h, kept at this temperature for 4 h, and cooled down to room temperature at the same rate. To obtain homogeneous samples, obtained ingots were crushed and milled into fine powders, compacted using a cold-press, heated in evacuated quartz ampules up to 773 K with a rate of 12 K/h, annealed at this temperature for 500 h, and quenched in cold water without breaking the containers.

### 2.2 Sintering

After the annealing process, the samples were crushed into fine powders by hand milling using an agate mortar and then densified by the pulsed electric current sintering (PECS) technique at 1073 K for 60 min in a 12.8 mm diameter graphite mold under an axial compressive stress of 40 MPa in an argon atmosphere. The heating and cooling rates were 70 and 50 K/min, respectively. Highly dense (ρ > 95% of crystallographic density) pellets with a diameter of 12.8 mm and a height of ∼2 mm were obtained and polished for transport property measurements.

### 2.3 Powder X-Ray Diffraction and Scanning Electron Microscopy

Phase identification was performed using a BRUKER D8 ADVANCE X-ray diffractometer using Cu *K*α radiation (λ = 1.5418 Å, Δ2Θ = 0.005^°^, and 2Θ range of 10–120^°^) with the Bragg–Brentano geometry. The Rietveld refinement of the crystal structure was carried out using WinCSD program package.^53^

For scanning electron microscopy (SEM) and energy-dispersive X-ray spectroscopy (EDS) analyses, the samples were embedded in conductive resin and subsequently polished, finally using 0.1 μm of diamond powder in a slurry. The analysis of the chemical composition was performed using SEM (JEOL JSM-6460LV scanning electron microscope) equipped with EDX spectroscopy.

### 2.4 Electrical and Thermal Transport Properties

The Seebeck coefficient *S* and electrical conductivity σ were measured using the commercial apparatus NETZSCH SBA 458 Nemesis. The measurements were performed in an argon flow at a temperature range of 298 to 673 K. The thermal diffusivity α_D_ was measured using a NETZSCH LFA 457 equipment, and the specific heat capacity *C*_p_ was estimated from the Dulong–Petit limit. The samples were first spray-coated with a thin layer of graphite to minimize errors from the emissivity of the material and laser beam reflection caused by a shiny pellet surface. Thermal conductivity was calculated using the equation κ = ρ*C*_p_α_D_, where ρ is the density obtained by the Archimedes principle at the disks from PECS. The uncertainty of the Seebeck coefficient and electrical conductivity measurements was 7 and 5%, respectively, whereas that of the thermal diffusivity measurements was 3%. The combined uncertainty for the determination of the TE figure of merit *ZT* was ∼20%.^54^ The Hall effect was investigated by applying the four-probe method in constant electric and magnetic fields (*H* = 0.9 *T*) and current through a sample of 50 mA. The uncertainty of Hall measurements was ∼10%. The speed of sound was measured at *T* = 298 K using the ultrasonic flaw detector Olympus Panametrics Epoch 3. The Vickers hardness of sintered samples was measured using a microhardness tester FM-700 developed by Future-Tech Corp., applying a load of 100 g. The optical absorbance spectra were measured using a Fourier transform infrared spectroscope (BRUKER VERTEX 70 V) at room temperature.

### 2.5 Electronic Band Structure Calculations

The electronic densities of states (DOSs) of Cu_2_MHf_3_S_8_ (M—Mn, Fe, Co, and Ni) were calculated using the Korringa–Kohn–Rostoker (KKR) method with the coherent potential approximation (CPA) that enables us to treat the chemical disorder induced by M substitution on the Hf site as a random atom distribution.^55,56^ In our calculations, the crystal potential was constructed within the local density approximation (LDA), employing the parameterization of Perdew and Wang for the exchange–correlation part.^57^ The position of the Fermi level *E*_F_ was determined accurately from the generalized Lloyd formula.^58^ For all the compositions, the experimental lattice parameters (Table 1) and atomic coordinates (Table 2), determined from Rietveld refinements against the powder X-ray diffraction (PXRD) data, were used. For well-converged crystal potential and atomic charges (below 1 meV and 10^–3^*e*, respectively), the total-, site-, and *l*-decomposed DOS (truncated at *l*_max_ = 2) were computed using a tetrahedron method for integration in the reciprocal *k*-space.^58^ The electronic band structure was also computed for all the investigated compounds in the framework of the complex energy KKR-CPA calculations, where the real part of the *E*(*k*) dispersion curves was extracted to plot electronic bands along high-symmetry directions in the *fcc* Brillouin zone.

**Table 1:** Results of the Crystal Structure Determination of the Cu_2_MHf_3_S_8_ (M—Mn, Fe, Co, and Ni) Compounds

	Cu_2_MnHf_3_S_8_	Cu_2_FeHf_3_S_8_	Cu_2_CoHf_3_S_8_	Cu_2_NiHf_3_S_8_
space group	*Fd*3̅*m* (no. 227)			
*a* (Å)	10.39759(3)	10.33404(3)	10.32084(2)	10.30074(2)
*V* (Å^3^)	1124.083(8)	1103.597(8)	1099.372(6)	1092.963(6)
number of atoms in a cell	56	56	56	56
calculated density (g/cm^3^)	5.78	5.95	6.07	6.08
absorption coefficient (1/cm)	773.1	802.0	822.0	733.4
radiation and wavelength	Cu *K*α 1.54185 Å			
diffractometer	BRUKER D8 ADVANCE			
mode of refinement	full profile			
number of atom sites	4			
number of free parameters	1			
2θ and sin θ/*γ* (max)	120.00 0.562			
*R*_I_	0.0520	0.0384	0.0244	0.0306
*R*_P_	0.0818	0.0823	0.0533	0.1000
scale factor	0.09818	0.13065	0.09678	0.12190

**Table 2:** Atomic Coordinates and Isotropic Temperature Displacement Parameters for the Cu_2_MHf_3_S_8_ (M—Mn, Fe, Co, and Ni) Compounds

atom	*x*/*a*	*y*/*b*	*z*/*c*	*B*_iso_×10^2^, Å^2^	N
**Cu**_**2**_**MnHf**_**3**_**S**_**8**_					
Cu	1/8	1/8	1/8	0.69(8)	8
Mn*	1/2	1/2	1/2	0.63(1)	16
Hf*	1/2	1/2	1/2	0.87(3)	16
S	0.7472(3)	0.7472(3)	0.7472(3)	0.67(6)	32
*—Occupancy: Mn—0.241(3) Mn; Hf—0.759(3) Hf					
**Cu**_**2**_**FeHf**_**3**_**S**_**8**_					
Cu	1/8	1/8	1/8	0.91(5)	8
Fe*	1/2	1/2	1/2	0.4(3)	16
Hf*	1/2	1/2	1/2	0.99(2)	16
S	0.7455(2)	0.7455(2)	0.7455(2)	0.78(4)	32
*—Occupancy: Fe—0.220(2) Fe; Hf—0.780(2) Hf					
**Cu**_**2**_**CoHf**_**3**_**S**_**8**_					
Cu	1/8	1/8	1/8	1.65(6)	8
Co*	1/2	1/2	1/2	0.2(2)	16
Hf*	1/2	1/2	1/2	0.89(2)	16
S	0.7444(2)	0.7444(2)	0.7444(2)	0.98(5)	32
*—Occupancy: Co—0.220(7) Co; Hf—0.780(3) Hf					
**Cu**_**2**_**NiHf**_**3**_**S**_**8**_					
Cu	1/8	1/8	1/8	1.03(5)	8
Ni*	1/2	1/2	1/2	0.53(14)	16
Hf*	1/2	1/2	1/2	0.97(2)	16
S	0.7437(1)	0.7437(1)	0.7437(1)	0.71(4)	32
*—Occupancy: Ni—0.228(6) Ni; Hf—0.772(2) Hf					

## 3 Results and Discussion

### 3.1 Crystal Structure and Microstructural Properties

The determination and refinement of the crystal structure of the Cu_2_MHf_3_S_8_ chalcogenide phases (M—Mn, Fe, Co, and Ni) were performed using X-ray powder diffraction. The diffraction patterns were indexed in cubic symmetry (SG *Fd*3̅*m*, Pearson symbol *cF*56). The conditions and results of the X-ray experiments are presented in Table 1. The analysis of *hkl* indices, reflections, and intensities indicated that the synthesized chalcogenide phases belong to the MgAl_2_O_4_^25^ structure type. The atomic coordinates of the Cu_7.38_Mn_4_Sn_12_S_32_^59^ compound were used as the starting computation model. The structural parameter refinement was performed by the Rietveld method using a gradual approximation of the calculated diffraction pattern profiles to the experimental patterns (Figure S1).

In Table 1, we can observe that the unit cell parameter of the Cu_2_MnHf_3_S_8_ compound is significantly higher than the unit cell for the other investigated thiospinels. It is connected with the variation of the ionic radii of transition metals (Mn, Fe, Co, and Ni). According to the data of Vainshtein et al.,^60^ the ionic radii of Mn^2+^ (0.91 Å) is significantly larger than the ion radii of Fe^2+^ (0.80 Å), Co^2+^ (0.78 Å), and Ni^2+^ (0.74 Å). Therefore, the compositional dependence of the unit cell parameter is in good agreement with the change of the M ionic radii for the investigated Cu_2_MHf_3_S_8_ thiospinels.

The refinement of coordinates and isotropic thermal displacement parameters of atoms (Table 2) yields satisfactory values of the fit factors and proves the validity of the model. A somewhat higher value of the B_iso_ for Cu atoms in the Co-containing specimen, compared to the other investigated samples, can be caused by the presence of specific Co atoms that occupy close octahedral voids. As it was reported for homologous Cu_2_CoTi_3_S_8_ using single-crystal data,^61^ the isotropic thermal displacement parameter of the Cu atom *U*_eq_ = 2.17 × 10^–2^ Å^2^ is almost two times higher than the corresponding value *U*_eq_ = 1.25 × 10^–2^ Å^2^ of Cu atoms in unsubstituted Cu_2_Ti_4_S_8_.^62^ Moreover, for the case of Cu_2_M_0.6_Ti_3.4_S_8_, it was reported that the increase of the isotropic thermal displacement parameter of the Cu atom in the series Mn–Fe–Co (*U*_eq_ = 1.32 × 10^–2^ Å^2^ for M = Mn, *U*_eq_ = 1.51 × 10^–2^ Å^2^ for M = Fe, and *U*_eq_ = 1.96 × 10^–2^ Å^2^ for M = Co).^61^ A similar tendency in the isotropic thermal displacement parameter of the Cu atom is observed in the case of our compounds (Table 2).

As the losses of copper or sulfur are highly expected during the high-temperature preparation of Cu-based sulfides, we took into account the different weighting schemes for the deviation of these elements during Rietveld refinement. Nevertheless, the best agreement between the experimental and calculated PXRD patterns corresponds to the site occupancy factor of 1.0 for these elements in the investigated samples. Therefore, we conclude that no losses of Cu and S during synthesis were observed.

**Figure 1:**
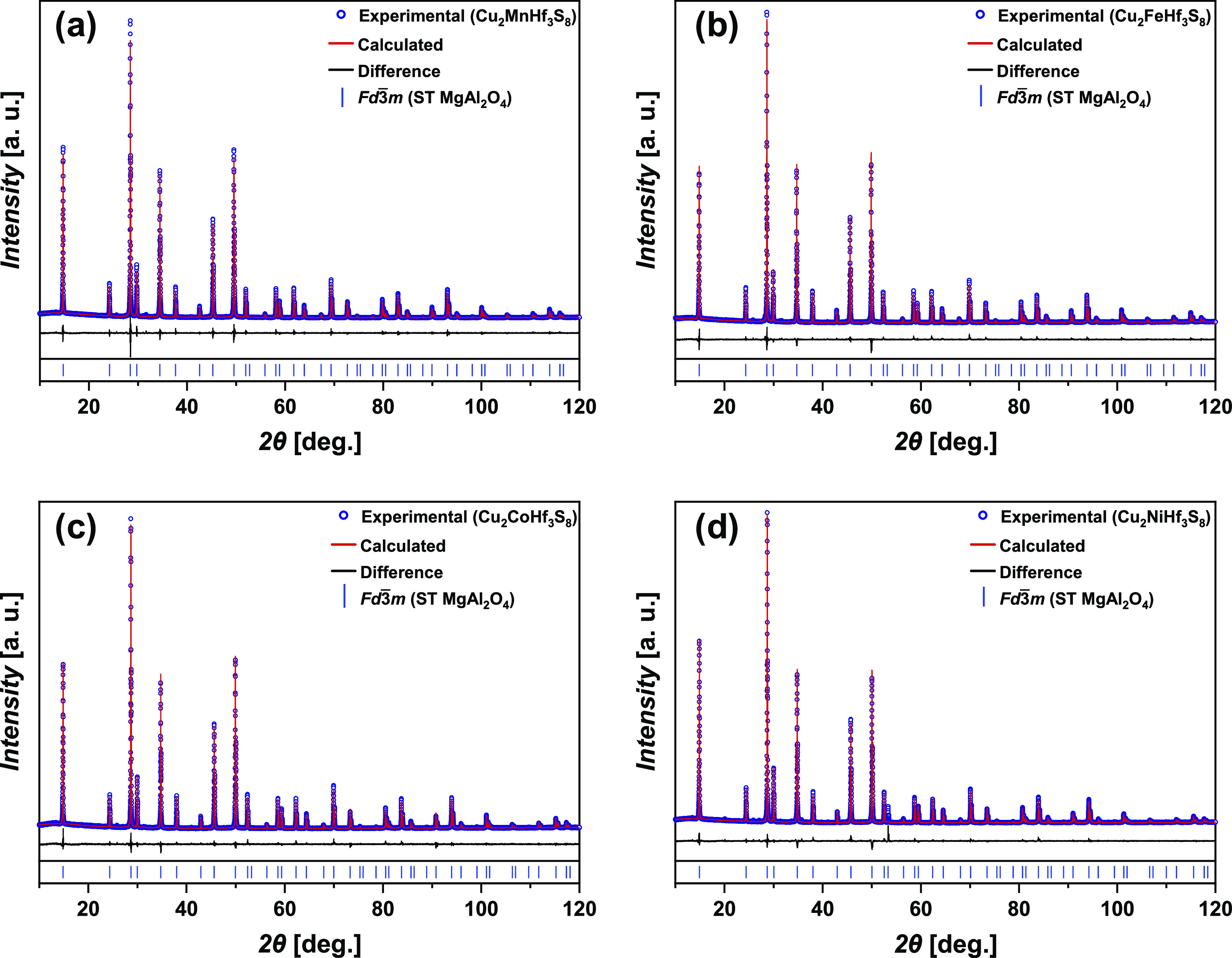
Results of the Rietveld refinement of the Cu_2_MHf_3_S_8_ (M—Mn, Fe, Co, and Ni) compounds.

The experimental and calculated diffraction patterns of Cu_2_MHf_3_S_8_ chalcogenides and their difference are shown in Figure 1a–d.

The results of the calculation of interatomic distances and coordination numbers of atoms in the Cu_2_MHf_3_S_8_ structures are presented in Table 3. The interatomic distances correlate well with the sums of the corresponding ionic radii.^63^

**Table 3:** Interatomic Distances (δ) and Coordination Numbers (C.N.) of Atoms in the Structure of Cu_2_MHf_3_S_8_ (M—Mn, Fe, Co, and Ni) Compounds

		Cu_2_MnHf_3_S_8_	Cu_2_FeHf_3_S_8_	Cu_2_CoHf_3_S_8_	Cu_2_NiHf_3_S_8_	
atoms		δ (Å)	δ (Å)	δ (Å)	δ (Å)	C.N.
Cu	– 4 S	2.302(2)	2.318(1)	2.334(2)	2.343(1)	4
M	– 6 S	2.571(2)	2.538(1)	2.524(2)	2.511(1)	6
Hf	– 6 S	2.571(2)	2.538(1)	2.524(2)	2.511(1)	6
S	– 1 Cu	2.302(2)	2.318(1)	2.334(2)	2.343(1)	4
	– 3 L^a^	2.571(2)	2.538(1)	2.524(2)	2.511(1)	

a *L* = M + Hf.

**Figure 2:**
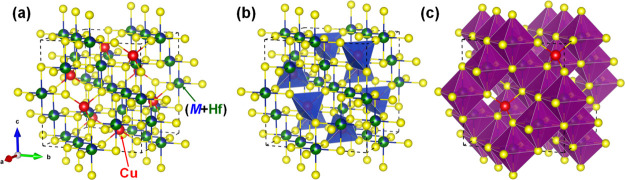
Unit cell (a), representation of the coordination environment of Cu atoms (b), and (M + Hf) atoms (c) for the Cu_2_MHf_3_S_8_ (M—Mn, Fe, Co, and Ni) crystal structure.

The structure of discovered compounds is based on the three-layer close packing of sulfur atoms. The nature of filling 1/2 octahedral voids with the statistical mixture of atoms *L* = M + Hf (M—Mn, Fe, Co, and Ni) and 1/8 tetrahedral voids with Cu, respectively, causes the differentiation of the unit cell in the structure into octants and the formation of smaller F-cubes. Cu atoms in such an eightfold cell are located in the tetrahedral surroundings of sulfur atoms (Figure 2).

The atoms of the statistical mixture are located in centrosymmetric sites and S atoms in monovariant sites on the third-order axes (Bravais lattice 3*m*). The first coordination sphere of sulfur is the tetrahedron. The cation–anion distances for the octahedral sites are averaged, and for Cu, the distances correspond to the sum of the tetrahedral radii. Quaternary copper-containing sulfides of transition 3*d* elements belong to the phases with mixed coordination. The ordered occupation of tetrahedral and octahedral positions in the structure attributes them to normal chalcogenide spinels.

**Figure 3:**
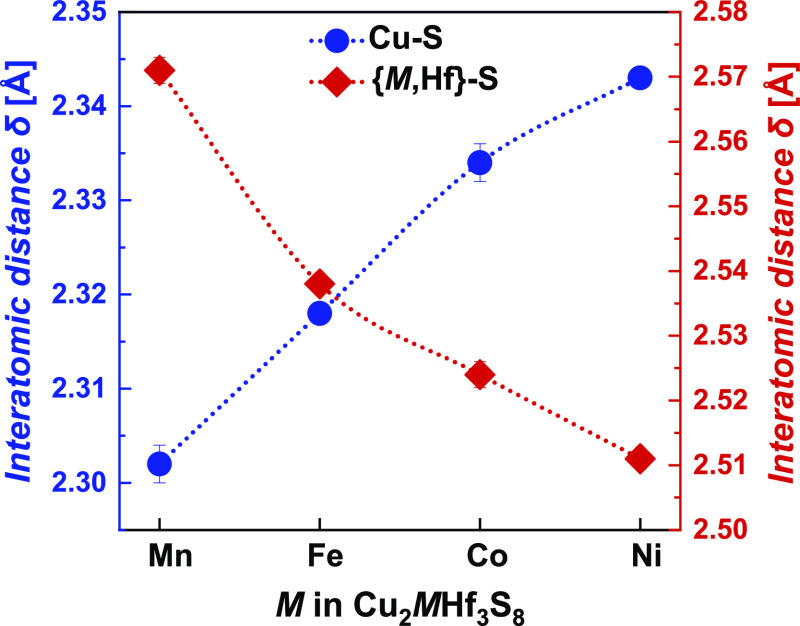
Change of the Cu–S and (M + Hf)–S interatomic distances for Cu_2_MHf_3_S_8_ compounds.

The gradual increase of the interatomic distance Cu–S and the decrease of the interatomic distance (M/Hf)–S are observed in the Mn → Fe → Co → Ni series (Figure 3). The latter is associated with the decrease of the ionic radius of M atoms in the series.

**Figure 4:**
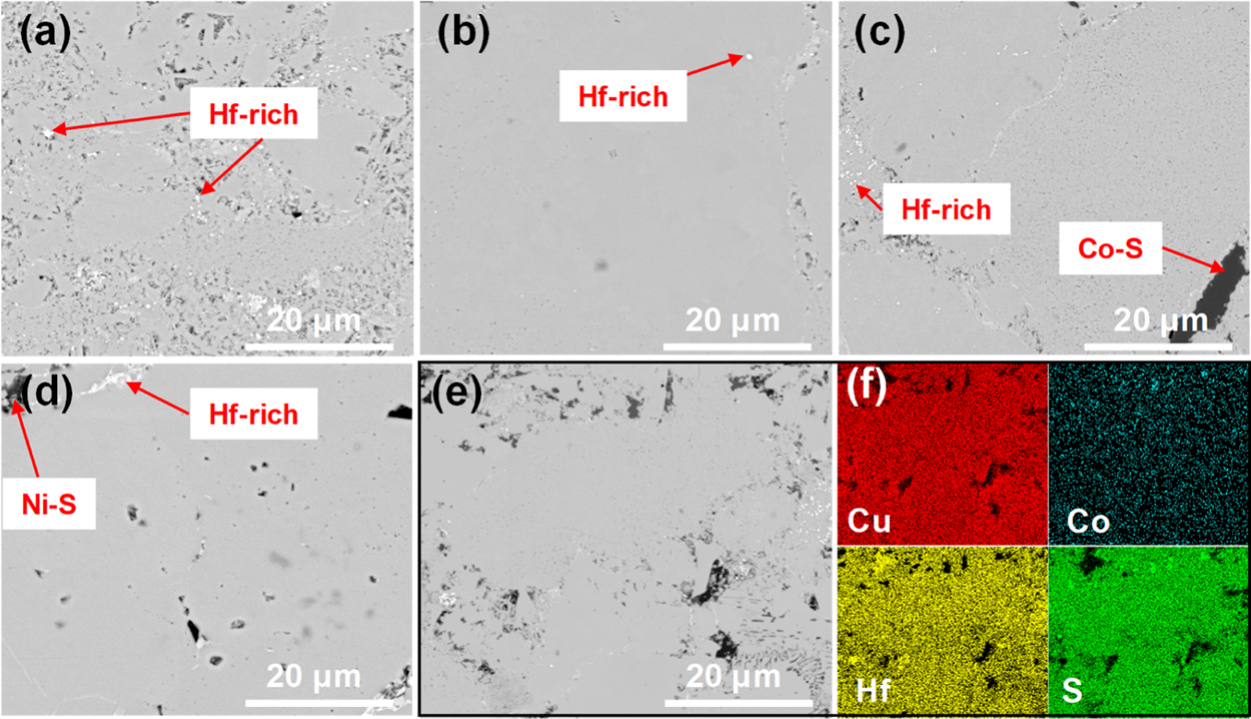
BSE images of Cu_2_MHf_3_S_8_ samples: (a) M—Mn, (b) M—Fe, (c) M—Co, and (d) M—Ni. BSE image of the Cu_2_CoHf_3_S_8_ sample (e) with EDS element mapping (f).

Figure 4 shows backscattered electron (BSE) images of Cu_2_MHf_3_S_8_ samples (M—Mn, Fe, Co, and Ni). All the samples were generally single phase, however, with a small presence of Hf-rich sub-micro precipitates. The Co- and Ni-containing samples (Figure 4c,d) additionally have some minor traces of Co–S and Ni–S phases. The chemical composition of the main phase for all the investigated samples was very close to the nominal composition, as determined by Rietveld refinements and EDS analysis. Co- and Ni-rich precipitates were found to have a chemical composition close to Co_9_S_8_ and Ni_9_S_8_, respectively, in agreement with PXRD data. The precise identification of the chemical composition of Hf-rich sub-micro precipitates was problematic due to their small dimensions. However, the amount of secondary phases is very small, therefore we do not expect any modification of physical properties due to these phases. Figure 4e,f shows EDS element distribution mapping, where only small agglomerations of Co-rich and Hf-rich phases were detected.

### 3.2 Electronic Transport Properties

The transport properties of Cu_2_MHf_3_S_8_ (M—Mn, Fe, Co, and Ni) specimens at *T* = 298 K are shown in Table 4. The absolute values of the carrier concentration *n*_H_ for all the studied compounds are in the range of 10^16^–10^17^ cm^–3^.

**Table 4:** Seebeck Coefficient *S*, Electrical Conductivity σ, Thermal Conductivity κ, Hall Carrier Concentration *n*_H_, and Carrier Mobility μ for the Cu_2_MHf_3_S_8_ Polycrystalline Samples at *T* = 298 K

compound	*S* [μV K^–1^]	σ [S m^–1^]	κ [W m^–1^ K^–1^]	*n*_H_ [cm^–3^]	μ [cm^2^ V^–1^ s^–1^]
Cu_2_MnHf_3_S_8_	357	0.031	1.15	4.0 × 10^16^ (*p*)	0.1
Cu_2_FeHf_3_S_8_	172	2.4	0.96	2.3 × 10^16^ (*p*)	6.5
Cu_2_CoHf_3_S_8_	–322	8.7	0.79	6.8 × 10^16^ (*n*)	8.0
Cu_2_NiHf_3_S_8_	–120	775	0.79	6.4 × 10^17^ (*n*)	75.7

As suggested by Kariya et al.,^27^ the charge balance for Cu_2_Hf_4_S_8_ can be written as Cu_2_^+^Hf^2+^Hf_3_^4+^S_8_^2–^ with two configurations of hafnium: Hf^2+^–5d^2^ and Hf^4+^–5d^0^. In our case, Hf^2+^ is substituted by M (M—Mn, Fe, Co, and Ni) in Cu_2_MHf_3_S_8_. Cu_2_MnHf_3_S_8_ possesses a very high positive Seebeck coefficient (357 μV K^–1^ at 298 K), which is typical for an intrinsic semiconductor with a low carrier concentration (4.0 × 10^16^ cm^–3^). The density functional theory (DFT) calculations of the electronic structure also confirm that *E*_F_ is lying in the valence band (VB) close to the band gap edge. Cu_2_FeHf_3_S_8_ shows a lower positive Seebeck coefficient with a lower concentration of holes of 2.3 × 10^16^ cm^–3^ compared with the Cu_2_MnHf_3_S_8_. The lower carrier concentration can be connected to the electrons introduced into the system due to an additional *d*-electron in Fe^2+^ (3d^6^) compared to Mn^2+^ (3d^5^). The lower Seebeck coefficient can originate from the effect of the minority carriers, which is highly probable in the undoped compounds with a narrow band gap. The DFT calculations also indicate that the *E*_F_ is tending to fall into the band gap, which is in line with the abovementioned explanation. Cu_2_CoHf_3_S_8_ shows a negative Seebeck coefficient with a low concentration of dominant charge carriers (electrons) of 6.8 × 10^16^ cm^–3^. This can be explained by the shift of the Fermi level toward the conduction band (CB) in contrast with the Cu_2_FeHf_3_S_8_ and Cu_2_MnHf_3_S_8_. This observation is also in agreement with the electron configuration of Co^2+^ (3d^7^). More *d*-electrons first cause the self-compensation of holes, and later change the material from *p*- to *n*-type. As a result, Cu_2_NiHf_3_S_8_ shows a lower negative Seebeck coefficient (−120 μV K^–1^). The dominant carriers are electrons, and the measured Hall concentration *n*_H_ = 6.4 × 10^17^ cm^–3^ is higher compared to the case of Cu_2_CoHf_3_S_8_, where *n*_H_ = 6.8 × 10^16^ cm^–3^_._ The larger number of *d*-electrons (3d^8^) shifts the Fermi level deeper into the CB, decreases the absolute value of the Seebeck coefficient, and enhances electrical conductivity. The performed analysis indicates that further steps (i.e., deviation from stoichiometry and doping) should be performed to tune the concentration to the optimal value for the maximum energy conversion efficiency,^50^ which can be the object of future studies.

**Figure 5:**
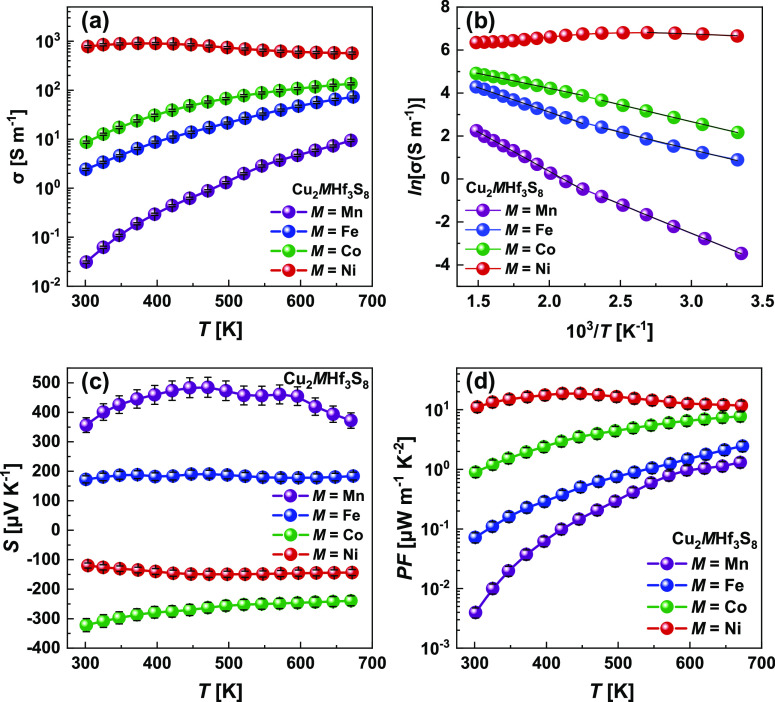
Electrical conductivity (a), Arrhenius plot of electrical conductivity (b), Seebeck coefficient (c), and TE power factor *PF* (d) for Cu_2_MHf_3_S_8_.

Figure 5 shows the electrical conductivity (panel a) and the Arrhenius plot of electrical conductivity (panel b) for Cu_2_MHf_3_S_8_ polycrystalline samples over the entire temperature range of 298–673 K. The electrical conductivity σ for the Cu_2_MnHf_3_S_8_ sample increased from ∼0.03 S m^–1^ at 298 K to ∼9.4 S m^–1^ at 673 K (Figure 5a). The electrical conductivity of Cu_2_FeHf_3_S_8_ and Cu_2_CoHf_3_S_8_ increase from ∼2.4 S m^–1^ at 298 K to ∼73 S m^–1^ at 673 K and from ∼8.7 S m^–1^ at 298 K to ∼135 S m^–1^ at 673 K for Fe- and Co-containing thiospinels, respectively. The electrical conductivity of Cu_2_NiHf_3_S_8_ shows the highest values and just slightly increases with temperature from ∼775 S m^–1^ at 298 K to ∼900 S m^–1^ at 373 K, and then decreases to ∼570 S m^–1^ at 673 K, showing weak metallic behavior (Figure 5a). The values of σ for Cu_2_MHf_3_S_8_ samples are lower than other Cu-based thiospinels, especially homologous Cu_2_MTi_3_S_8_ (M = Mn, Fe, Co, and Ni), studied by Hashikuni et al.,^48^ which shows electrical conductivity in the range of ∼4.3 × 10^4^–1.1 × 10^5^ S m^–1^ at 298 K. However, the obtained values of σ are similar to Sn-based thiospinels, especially the Cu_2_CoSn_3_S_8_ compound, which increases from ∼200 S m^–1^ at 323 K to ∼700 S m^–1^ at 673 K.^34^

Electrical conductivity activation energies *E*_a_, estimated from the Arrhenius plot of electrical conductivity (Figure 5b) for Cu_2_MHf_3_S_8_ polycrystalline samples are given in Table 5. The values of *E*_a_ for Cu_2_MnHf_3_S_8_ are in the range of 0.46–0.63 eV, which is in good agreement with the lowest electrical conductivity and the highest Seebeck coefficient for this sample. The activation energies for Cu_2_FeHf_3_S_8_ and Cu_2_CoHf_3_S_8_ are higher and vary in a range of 0.24–0.39 eV. The values of *E*_a_ for Cu_2_NiHf_3_S_8_ are very small at low temperatures, probably due to hopping conductivity or the presence of some in-gap states. Considering the low values of the activation energies, we can conclude that the poor electrical conductivity observed for the investigated Cu_2_MHf_3_S_8_ alloys originates from the low charge carrier concentration.

**Table 5:** Activation Energy *E*_a_ in the Given Temperature Range for Studied Thiospinels

compound	*E*_a_ [eV]	temp. range [K]
Cu_2_MnHf_3_S_8_	0.46(1)	298–423
	0.63(1)	448–623
Cu_2_FeHf_3_S_8_	0.28(1)	298–423
	0.39(1)	448–673
Cu_2_CoHf_3_S_8_	0.27(1)	298–423
	0.24(1)	448–673
Cu_2_NiHf_3_S_8_	0.06(1)	298–373

Figure 5c represents the measured Seebeck coefficient for Cu_2_MHf_3_S_8_ polycrystalline samples prepared by the PECS technique at 1073 K. Two samples containing manganese and iron possess a positive Seebeck coefficient *S* throughout the entire temperature range, indicating that holes are the dominant carriers. The Seebeck coefficient for the pristine Cu_2_MnHf_3_S_8_ is monotonously increasing from 357 to 485 μV K^–1^ at 473 K and then decreases to 372 μV K^–1^ at 673 K due to the effect of minority carriers. The Seebeck coefficient for pristine Cu_2_FeHf_3_S_8_ shows roughly temperature-independent behavior, with values in the range of 170–190 μV K^–1^. The different temperature gradients of *S* for these two samples can be explained by the different band structures of the investigated compounds and differences in the Hall carrier concentration. Cobalt- and nickel-containing materials possess a negative Seebeck coefficient over the entire temperature range, indicating that electrons are the dominant charge carriers. The Seebeck coefficient for pristine Cu_2_CoHf_3_S_8_ shows a decreasing tendency from −322 to −239 μV/K and is much higher compared with the Seebeck coefficient of Cu_2_NiHf_3_S_8_ (from −120 to −150 μV K^–1^) at the temperature range of 298–673 K. The low Seebeck coefficient accompanied by the low carrier concentration can be connected with the compensation effect of majority carriers (electrons) by the minority carriers (holes) in Cu_2_NiHf_3_S_8_.

Based on the measured *S* and σ, the power factors (*PF* = *S*^2^σ) of all the studied Cu_2_MHf_3_S_8_ polycrystalline samples are calculated and presented in Figure 5d. Because of the increase of electrical conductivity in the series Mn–Fe–Co–Ni in a few orders of magnitude and simultaneously with considerably high Seebeck coefficients,^64^ the Cu_2_NiHf_3_S_8_ sample showed the highest values of *PF* over the whole temperature range. The heating/cooling cycles of the electronic transport properties for the investigated samples are shown in Figure S2 (Supporting Information). The electrical conductivity, Seebeck coefficient, and power factor are repeatable for all the materials, except Cu_2_MnHf_3_S_8,_ where the properties disagreed under heating and cooling. Such a tendency for this sample can be related to some chemical composition change or non-repeatable growth of intrinsic carrier concentration, as it was also observed for other thiospinels^42,44^ and some other chalcogenides.^65−67^

**Figure 6:**
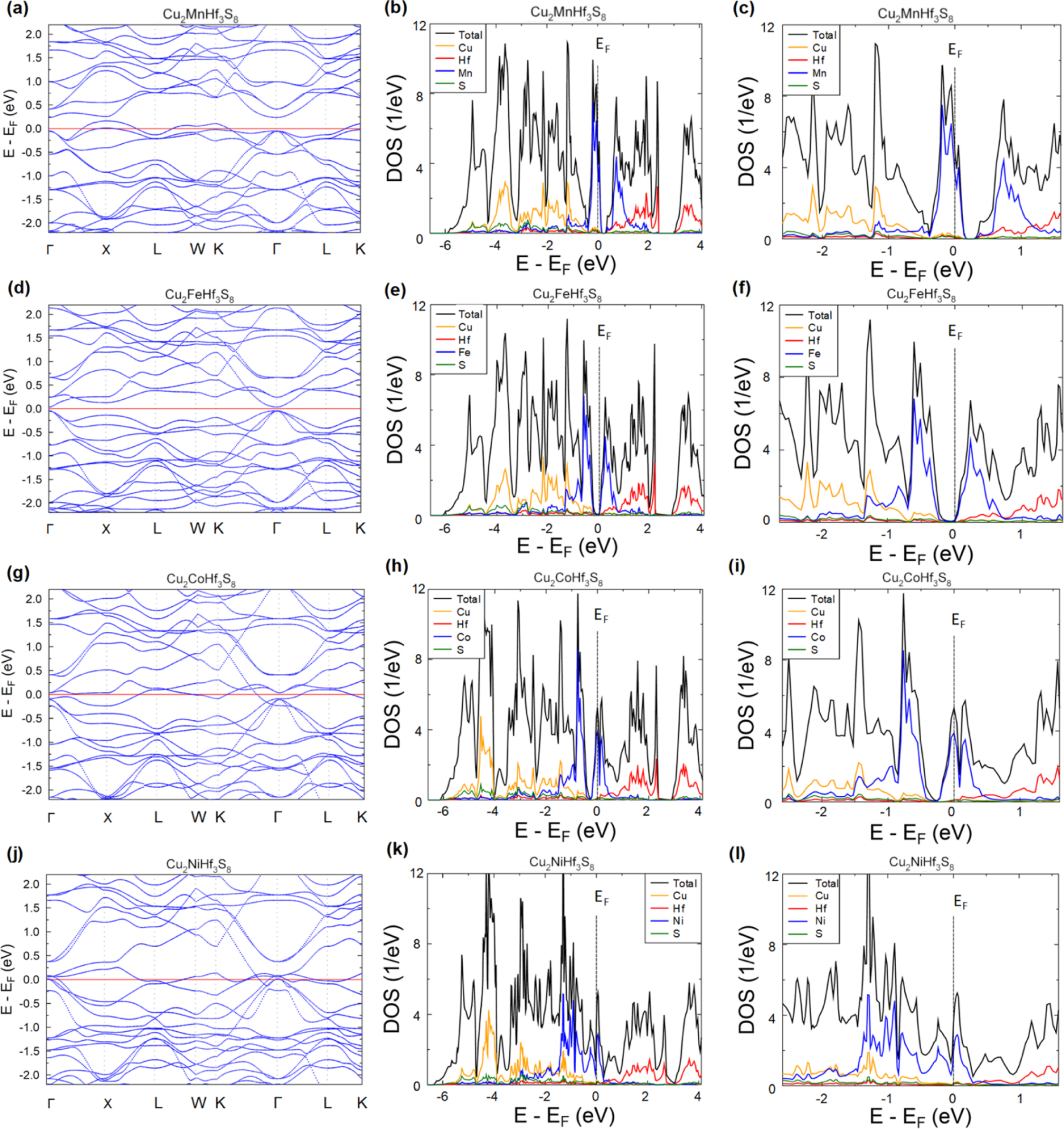
Electronic dispersion curves *E*(**k**) along the high-symmetry *fcc* Brillouin zone (left panel) and total- and site-decomposed DOS (middle panel) and DOS zoomed near *E*_F_ (right panel) for Cu_2_MHf_3_S_8_ thiospinels: M = Mn (a–c); M = Fe (d–f); M = Co (g–i); and M = Ni (j–l) from the non-spin-polarized KKR-CPA calculations. The Fermi level was set to zero (*E*_F_ = 0).

Electronic structure calculations using the KKR-CPA method were performed for the studied Cu_2_MHf_3_S_8_ (M—Mn, Fe, Co, and Ni) thiospinel compounds to have an insight into measured electrical transport properties strongly varying with M substitution. Accordingly, Figure 6a–l presents the comparison of total- and site-decomposed DOS and electronic dispersion curves E(***k***) for Cu_2_MnHf_3_S_8_ as Cu_2_FeHf_3_S_8_, Cu_2_CoHf_3_S_8_, and Cu_2_NiHf_3_S_8_ alloys. Looking at the DOSs of these systems, one can distinguish a common feature, that is, the appearance of a deep minimum or an energy gap in the electronic spectra (appearing around the Fermi level), which divides the block of the strongly hybridized *d*-states of transition metal atoms Cu, Hf, and M and the *p*-states of S into valence-like and conduction-like bands. Inspecting in more detail the dispersion curves, one can notice that the energy gap (DOS minimum) tends to be formed above the 45th VB (enabling to accommodate 90 electrons), if accounting for eight bands (16 electrons *s*-symmetry originating from S) lying c.a. 6 eV below the valence edge located at about energy −6 eV. Hence, the visible *p*–*d* block of valence states consists of 37 bands accommodating 74 electrons.

As mentioned above, the relatively open structure of the Cu_2_MHf_3_S_8_ thiospinels with variable interatomic distances and specific atomic coordination leads to the formation of tetrahedral bonds and *sp*^3^ hybridization around *p*-elements, favoring bonding between *d*-metals. The propensity of some systems to form the energy gap at a specific number of electrons (VEC—valence electron counts) can be compared to, for example, ternary half-Heusler systems, crystallizing in a similar *F*4̅3*m* structure and exhibiting semiconducting-like properties for VEC = 18. In the case of the thiospinel structure, it appears to have 90 valence electrons calculated for the primitive unit cell (or per chemical formula). In particular, the VEC condition is satisfied in Cu_2_FeHf_3_S_8_, where VEC = 2 × 11e (Cu) + 3 × 4e (Hf) + 1 × 8e (Fe) + 8 × 6e (S) = 90, and as a consequence, the Fermi level falls into the energy gap (or deep DOS minimum), responsible for semiconducting-like transport behaviors. Bearing in mind such electronic structure features of Cu_2_MHf_3_S_8_ thiospinels, the variety of electron transport behaviors with different M elements can be partly interpreted in terms of the number of electrons in the system.

Hence, Cu_2_MnHf_3_S_8_ also has a narrow band gap in the X point of the Brillouin zone, but due to one electron less in the system with respect to the VEC = 90 criterion, the Fermi level is located in the VB in agreement with measured *p*-type conductivity for this compound. As aforementioned, Cu_2_FeHf_3_S_8_ also has a very narrow band gap, however, the Γ point and Fermi level are shifted up to the band gap due to the additional *d*-electron of Fe^2+^ in comparison with Mn^2+^. Furthermore, the addition of d-electrons in the case of Cu_2_MHf_3_S_8_ (M—Co and Ni) makes them more metallic-like alloys (semimetals) with the Fermi level located in the CB, which is in agreement with the observed *n*-type conductivity for these compounds. The KKR-CPA results of the Cu_2_NiHf_3_S_8_ compound suggest that its ground state is metallic, which correlates well with the experimentally measured metallic-like character of the temperature-dependent electrical conductivity and the low Seebeck coefficient. The partial DOS shows that the VB and CB near *E*_F_, are mainly built up by M–3*d* states in Cu_2_MHf_3_S_8_ (M—Mn, Fe, Co, and Ni) compounds. That is why the substitution of the M atom in Cu_2_MHf_3_S_8_ has such a strong effect on electronic transport, observed during measurements. This observation also suggests that the substitution of M atoms can be the most effective way to modify the band structure for the investigated alloys. All the compounds show a multivalley band structure near the *E*_F_, however, the low values of electrical conductivity can be explained by the undesirable intervalley scattering of electrons. In contrast with Cu_2_MTi_3_S_8_ compounds, where mostly Ti forms states near the *E*_F_,^48^ Hf has a very small contribution in this region in Cu_2_MHf_3_S_8_ thiospinels. This can also be a reason for the low electrical conductivity of Cu_2_MHf_3_S_8_ thiospinels in comparison to Cu_2_MTi_3_S_8_ because the [M/Ti(Hf)]S_6_ network bears the electrical conduction in such kinds of compounds, as suggested in ref. (48)

Because of the LDA used, we expect that the calculated band gap energy is underestimated. Hence, the evolution of the electron structure of Cu_2_MHf_3_S_8_, as obtained from the KKR-CPA calculations, suggests the change in electrical conductivity from hole-like to electron-like and in thermopower from positive to negative with an increasing number of electrons (M = Mn, Fe, Co, and Ni), should be treated more qualitatively than quantitatively. It can be assumed that extending first principles calculations to the LDA + U approach, with extra repulsion on-site term U on transition metal elements, should be used to better explain the semiconducting character of the electrical conductivity measured for M = Mn and M = Co. Our KKR-CPA-LDA calculations rather indicate the proximity of the band gap (or the pseudogap) and the Fermi level lying on a strongly varying DOS slope. So that, the main goal of KKR(CPA) calculations in this work was to observe the evolution of the band structure in Cu_2_MHf_3_S_8_ compounds, not to provide the precise values of the *E*_g_. In the investigated series Mn–Fe–Co–Ni, it was found to have a decrease in conduction activation energies in agreement with such a tendency from KKR(CPA) calculations and with the literature reports for other similar thiospinels.^42^

The infrared absorption spectroscopy measurements have been performed to estimate the optical band gap for the investigated materials (Figure S3). From the dependence of optical absorption spectra versus photon energy, we observe at least two absorption edges in line with the complex electronic band structure of the investigated compounds. The measured smallest direct transitions correspond to 0.32 eV for the Cu_2_MnHf_3_S_8_ and 0.22 eV for Cu_2_FeHf_3_S_8_, Cu_2_CoHf_3_S_8_, and Cu_2_NiHf_3_S_8_ materials. The estimated band gaps are correlated with the activation energies calculated using temperature-dependent electrical conductivity. The values of *E*_g_ are also roughly consistent with the estimates obtained using the DFT calculations, where the decrease of the band gap can be detected; however, the LDA values of the *E*_g_ are lower compared with the optical data.^68^

**Figure 7:**
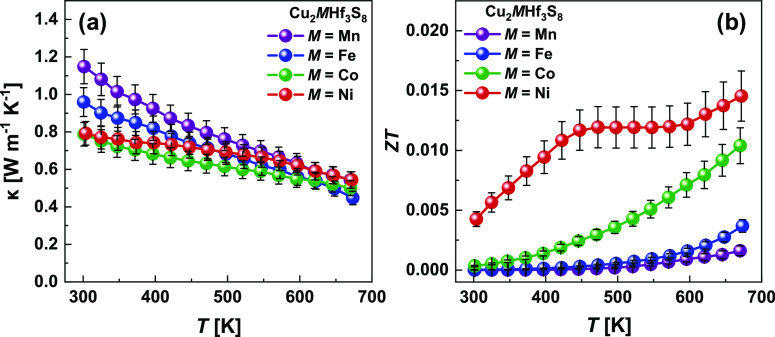
Thermal conductivity (a) and dimensionless figure of merit *ZT* (b) as a function of temperature for Cu_2_MHf_3_S_8_ polycrystalline samples.

Figure 7a shows the total thermal conductivity (κ) of the studied Cu_2_MHf_3_S_8_ (M—Mn, Fe, Co, and Ni) samples after sintering. All specimens possess very low thermal conductivities, in the range of 0.79–1.15 W m^–1^ K^–1^ at 298 K, decreasing to 0.45–0.54 W m^–1^ K^–1^ at 673 K, which are among the lowest values observed in spinel-type materials. In our case, the total κ is mainly contributed by the lattice thermal conductivity (κ_lat_) due to the very low electrical conductivity of the investigated samples. The origin of the remarkable reduction in thermal conductivity observed for the investigated alloys is discussed in the following section.

Combining the measured *S*, σ, and κ, the TE figure of merit (*ZT*) of the Cu_2_MHf_3_S_8_ polycrystalline samples are calculated and shown in Figure 7b. The beneficial effects of lower κ, however, have been negated by the poor *PF*, thus resulting in a low figure of merit *ZT* value of ∼0.015 at 673 K for the Cu_2_NiHf_3_S_8_ compound. Nevertheless, the highest PF and the low lattice thermal conductivity of Cu_2_NiHf_3_S_8_, makes this compound the most interesting for further investigation and tuning of carrier concentration, which can significantly improve the TE figure of merit.

The good TE performance requires a high power factor and low lattice thermal conductivity. If the κ for the investigated alloys shows very low values, the power factor is rather moderated, mainly due to the unoptimized carrier concentration. Therefore, to obtain a higher TE figure of merit *ZT* for the investigated thiospinels, the carrier concentration must be increased through proper doping or deviation from stoichiometry.

### 3.3 Origins of the Low Thermal Conductivity

Recent work by Hashikuni et al. shows that Cu_2_MTi_3_S_8_ (M—Mn, Fe, Co, and Ni) spinels possess much higher κ_lat_ ∼ 1.4–2.3 W m^–1^ K^–1^ at 298 K^48^ compared to the investigated Cu_2_MHf_3_S_8_ materials (κ_lat_ ∼ 0.79–1.15 W m^–1^ K^–1^ at 298 K). The explanation of this issue can be connected with the significant mass difference between Hf and M (M—Mn, Fe, Co, and Ni) compared to Ti vs. M. In this case, the large phonon scattering on point defects is expected, which may cause a reduction of the lattice thermal conductivity. On the other hand, the aforementioned features of the crystal structure, particularly the mixed occupation of octahedral voids by Hf and M, and the occupation of only three of six octahedra in the structure, provoke an increase in bond anharmonicity, which has recently been reported as one of the most powerful instruments for disturbing phonon transport.^69^

To shed some light on the measured thermal conductivity of the investigated alloys, we conjugated the ultrasonic measurements of longitudinal and transverse sound velocities at room temperature with the theoretical calculations based on the Callaway approach. The measured data of the longitudinal *v*_l_ and transverse *v*_t_, velocity and results of the calculations of the average velocities *v*_m_, Debye temperatures Θ_D_, the Poisson ratio ν, Grüneisen parameter γ, bulk modulus *B*, Young modulus *E*, phonon mean free path *l*_ph_, and the minimum thermal conductivity κ_glass_ and κ_diff_ for Cu_2_MHf_3_S_8_ (M—Mn, Fe, Co, and Ni) samples investigated in this work are shown in Table 6.

**Table 6:** Elastic and Thermal Transport Properties for Cu_2_MHf_3_S_8_ (M—Mn, Fe, Co, and Ni) Thiospinels

compound	*v*_l_, m/s	*v*_t_, m/s	*v*_m_, m/s	Θ_D_, K	ν	γ	*B*, GPa	*E*, GPa	*l*_ph_, Å	κ_glass_, W/(m K)	κ_diff_, W/(m K)
Cu_2_MnHf_3_S_8_	4094	2209	2466	263.8	0.29	1.74	55.0	49.5	6.95	0.64	0.40
Cu_2_FeHf_3_S_8_	4237	2229	2493	270.2	0.31	1.83	63.2	56.9	5.56	0.66	0.42
Cu_2_CoHf_3_S_8_	4072	2172	2427	261.3	0.30	1.78	56.5	50.8	4.29	0.64	0.41
Cu_2_NiHf_3_S_8_	3712	2009	2242	240.2	0.29	1.73	45.5	40.9	6.53	0.59	0.37

The measured speed of sound shows relatively high values compared to the other well-established TE materials, that is, PbTe,^69^ Bi_2_Te_3_,^70^ and GeTe.^3^ This may indicate that scattering of acoustic phonons is not the dominant mechanism that affects the phonon transport in the investigated materials.^71^ On the other hand, the quite high difference between the longitudinal *v*_l_ and transverse *v*_t_ speeds of sound causes the high Grüneisen parameters for the investigated materials. Such high values of γ, ranging from 1.73 to 1.83 (Table 6), are in line with the aforementioned hypothesis of the high degree of bond anharmonicity in the investigated compounds. Moreover, using inelastic neutron scattering, the anharmonicity has been experimentally demonstrated to be at the origin of the low lattice thermal conductivity in other sulfur-based compounds, for example, tetrahedrites.^72^ As shown in Figure 2, the studied structures are characterized by the (M + Hf)S_6_ octahedra. Introducing a heavier atom Hf instead of Ti or Sn in octahedra is evidently responsible for the evolution of bond anharmonicity.^69,73^ These lead to a very short phonon mean free path *l*_ph_ ∼4–7 Å that is about twice shorter than lattice parameters and can be a dominant mechanism responsible for disturbed phonon transport in our compounds. On the other hand, copper atoms form CuS_4_ tetrahedra with strong atomic interactions, which contribution to phonon scattering is rather small.

**Figure 8:**
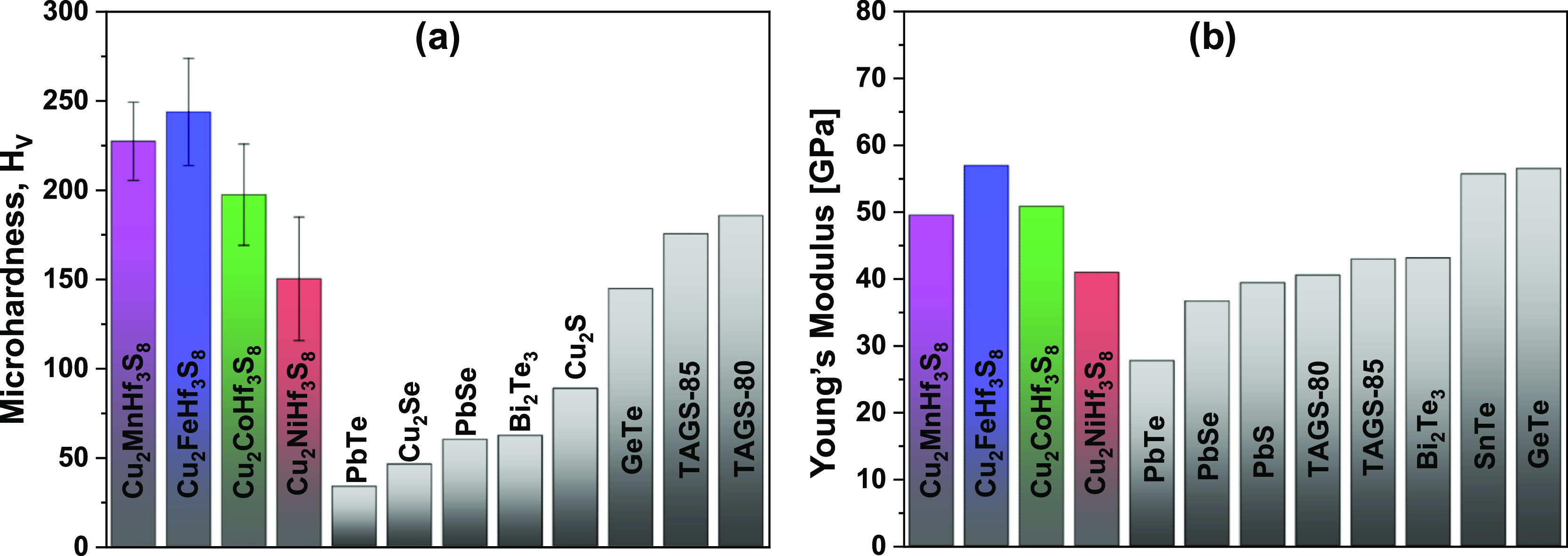
Microhardness of Cu_2_MHf_3_S_8_ samples in comparison to other conventional TE materials (a). Young’s modulus of Cu_2_MHf_3_S_8_ samples compared with the literature data (b).^74−76^

To examine the effect of M substitution on the mechanical properties of Cu_2_MHf_3_S_8_ samples, Vickers microhardness measurements were carried out, as shown in Figure 8a. In the series Mn–Fe–Co–Ni, the Vickers microhardness is generally decreasing; however, the Fe-containing sample shows the maximum value. On the other hand, such a compositional dependence of the measured microhardness excellently correlates with Young’s modulus determined from ultrasonic measurements, as shown in Figure 8b. Cu_2_MHf_3_S_8_ samples exhibit superior Young’s modulus and Vickers hardness, which is comparable (or even higher) to other well-established TE materials, suggesting their good mechanical stability.

We also calculated the “minimum thermal conductivity” using Cahill’s formulation^77^
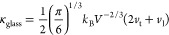
1where *V* is the average volume per atom calculated from the refined lattice parameters and *k*_B_ is the Boltzmann constant. As the calculated values of the lattice thermal conductivity were found to be higher than the experimental points, the diffusive-based minimum of the thermal conductivity κ_diff_ was calculated using the formalism proposed by Agne et al.^78^
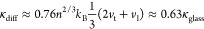
2

We see that the measured samples showed κ values that are close to the minimum of the thermal conductivity estimated from the assumption of diffuson-mediated thermal transport.^78^ The performed analysis also suggests that the complexity of the crystal structure caused by Hf atoms, that are introduced into the octahedral voids leads to a remarkable reduction of lattice thermal conductivity.^79^ Moreover, the abundance of structural voids and cationic disorder on the Hf/M site further contributes to the lowering of lattice thermal conductivity, as it was also recently shown for other Cu-based chalcogenides, for example, Cu_2_SnS_3_^80−82^ and colusites.^83−85^ However, the realistic mechanism that can describe the origin of the observed ultralow κ_lat_ is still unclear.

To further understand the role of Hf on the thermal transport properties of Cu_2_MHf_3_S_8_ (M—Mn, Fe, Co, and Ni), we used the Callaway approach.^86^ In this case, the phonon relaxation time (τ_c_) is calculated using contributions related to scattering on point defects^86^ and four-phonon processes^87^
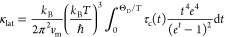
3
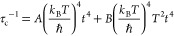
4where, ℏ = *h*/(2π) and *t* = ℏω/(*k*_B_*T*), A and B are adjustable fitting parameters related to point defect scattering and four-phonon Umklapp scattering processes, respectively. The materials considered in this work have large values of the Grüneisen parameter, this indicating high anharmonicity, which was identified as a factor that promotes the influence of the four-phonon Umklapp scattering processes.^87,88^ The use of the four-phonon Umklapp process allowed for much better fit quality over the fitting with the three-phonon Umklapp process and for obtaining smaller uncertainties of the fitted variables.

All the investigated lattice thermal conductivity dependences were reasonably well fitted by the Callaway model. The fitting parameters A and B (Table 7) quantify the strength of phonon scattering on point defects^89^ and four-phonon scattering processes,^87^ respectively. Therefore, their comparative analysis might provide a deeper insight into the origins of lattice thermal conductivity reduction.

**Table 7:** Fitted A and B Parameters of Cu_2_MX_3_S_8_ in the Callaway Model for the Calculation of Lattice Thermal Conductivity [A = (10^–38^ s^3^) and B = (10^–43^ s^3^ K^–2^)]

		(I)					
	*X*→	Hf		Sn		Ti	
	**M↓**	**A**	**B**	**A**	**B**	**A**	**B**
(IIa)	**Mn**	1.465(20)	0.613(11)			1.305(18)	0.145(08)
	**Fe**	1.737(30)	0.609(16)				
(III)	**Co**	2.646(33)	0.449(15)	2.240(99)	0.058(36)	1.099(20)	0.143(08)
(IIb)	**Ni**	3.091(43)	0.378(19)			0.908(19)	0.144(09)

**Figure 9:**
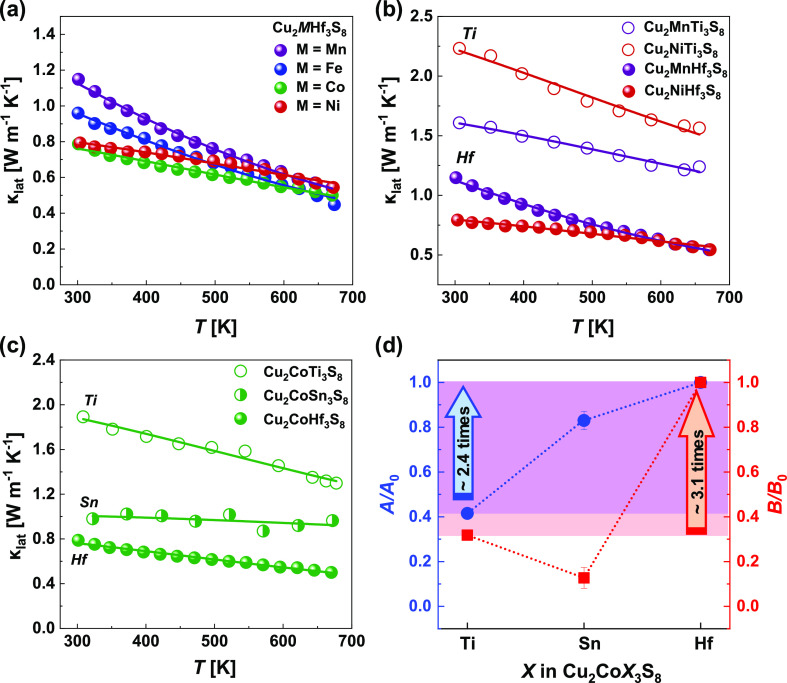
(a) Lattice thermal conductivity for Cu_2_MHf_3_S_8_ (M—Mn, Fe, Co, and Ni). (b) Comparison of lattice thermal conductivity of Hf-contained samples with the Ti-contained ones. (c) Comparison of lattice thermal conductivity for Cu_2_CoX_3_S_8_ (X = Hf, Sn, and Ti). The reduction of lattice thermal conductivity for the series Ti → Sn → Hf is obvious. In panels a–c, points indicate experimental data received in the present work or taken from refs.,^31,34,48^ lines correspond to the calculations using the Callaway approach. (d) Fitting parameters A and B, which were used for the calculation of lattice thermal conductivity by the Callaway approach for the Cu_2_CoX_3_S_8_ (X = Hf, Sn, and Ti). Parameter A quantifies the strength of phonon scattering on point defects^89^ and parameter B denotes the four-phonon scattering processes.^87^ Therefore, the reduction of the lattice thermal conductivity for investigated samples is coming from both mechanisms; however, the latter one is dominant.

In the case of Hf-based samples investigated in this work (Figure 9a, Table 7I), the A parameter increases in the Mn → Fe → Co → Ni series, which could be related to the observed decrease in atomic radius in this series, and thus an increase in the difference between the atomic radii of Hf and X, which increases strain in the material. At the same time, parameter B decreases in the Mn → Fe → Co → Ni series, indicating a slight reduction of the anharmonic scattering with the decrease of interatomic distances (M/Hf)–S.

A much more interesting observation has occurred after the comparison of the lattice thermal conductivity of spinels with Hf (this work) and Sn^34^ and Ti^31,48^ (Figure 9b–d, Table 7IIa,b). The Hf-induced large atom mass and size differences significantly increase the strength of phonon scattering on point defects (A parameter) by more than ∼2.3 times. However, the B parameter in the case of Hf-based spinels investigated in this work shows even more than ∼3.1 times difference compared with the Sn- and Ti-based compounds, suggesting a significantly larger effect of four-phonon scattering. As the four-phonon scattering is largely defined by bond anharmonicity, we can conclude that this effect could be determinative for the phonon transport in the Cu_2_MHf_3_S_8_ (M = Mn, Fe, Co, and Ni) spinels.

## 4 Conclusions

Thermal transport engineering through understanding the role of structural properties is among the newest ways to increase the functionality of TE materials. In this work, we successfully synthesized and investigated the crystal structure and electronic and band structure properties, and established the TE performance for four new Cu_2_MHf_3_S_8_ (M—Mn, Fe, Co, and Ni) thiospinels. It was found, that the discovered compounds crystallize in the space group *Fd*3̅*m* (No 227, Pearson symbol *cF*56) with a large number of atoms in the unit cell [*N* = 56 per cubic cell with *a* from 10.398 Å (for Mn) to 10.301 Å (for Ni)].

The DFT calculations using the KKR-CPA method suggest that the main contribution to the total density of electronic states, close to the Fermi energy, comes from M–3*d* electrons (Mn, Fe, Co, and Ni). Besides, the computed electronic band structure features near *E*_F_ reveal an apparent correlation between the number of valence electrons in the system and its strongly changing physical properties. Therefore, the significant modification of the electronic transport properties for the investigated Cu_2_MHf_3_S_8_ (M—Mn, Fe, Co, and Ni) thiospinels is expected due to substitution or partial substitution of these atoms. In line with the DFT results, the decrease in the activation energies and the transition from p- to n-type conductivity were observed in the Mn–Fe–Co–Ni series. The best dimensionless TE figure of merit *ZT* in this work was determined for the Cu_2_NiHf_3_S_8_ thiospinel due to the highest power factor and low thermal conductivity.

If the electrical transport properties for the investigated compounds are moderated mainly due to low electrical conductivity, the thermal conductivity shows very low values (as low as 0.50 W m^–1^ K^–1^ at 673 K for Cu_2_CoHf_3_S_8_) compared to the other reported thiospinels. The origins of such low thermal conductivity are connected with the introduction of Hf to the structure and particularities of the crystal lattice. On the one hand, the heavy Hf atoms cause a large mass difference effect between Hf and the other atoms in the structure, which reduces the lattice thermal conductivity. On the other hand, the atoms of Hf are located in the (Hf/M)S_6_ octahedral voids, and only half of these voids are occupied. Such a combination of crystal structure properties promotes large bond anharmonicity. The estimated from the speed of sound measurements high values of the Grüneisen parameters γ (1.73–1.83) are in line with this statement. Moreover, using the Callaway approach, we were able to evaluate that the contribution of the bond anharmonicity to the reduction of the thermal conductivity is much larger than the mass difference effect for the investigated alloys. This intriguing finding suggests Cu_2_MHf_3_S_8_ (M—Mn, Fe, Co, and Ni) thiospinels as novel and promising functional materials with intrinsically low lattice thermal conductivity. Moreover, the work offers bond anharmonicity as a powerful instrument for disturbing phonon transport in TE materials, particularly in lightweight thiospinels.

## Supporting Information Available

Cu_2_CoHf_3_S_8_ (CIF)
Cu_2_FeHf_3_S_8_ (CIF)
Cu_2_MnHf_3_S_8_ (CIF)
Cu_2_NiHf_3_S_8_ (CIF)
PXRD patterns of samples after synthesis and pellets after measurements; heating and cooling data of electronic transport properties; optical absorption spectra; and details of the elastic and thermal transport properties calculations (PDF)

## Supplementary Material

cm1c03593_si_001.cif

cm1c03593_si_002.cif

cm1c03593_si_003.cif

cm1c03593_si_004.cif
