# Supplementary material for: Crystal Structure and Thermoelectric Properties of Novel Quaternary Cu2MHf3S8 (M—Mn, Fe, Co, and Ni) Thiospinels with Low Thermal Conductivity
Source: Chem Mater. 2022 Feb 15;34(5):2146–60. doi: 10.1021/acs.chemmater.1c03593 (PMC8910496; doi:10.1021/acs.chemmater.1c03593)
Supplement: Supplementary file 5 — cm1c03593_si_005.pdf [file cm1c03593_si_005.pdf]

Supporting Information

Crystal structure and thermoelectric properties of novel quaternary $Cu_2MHf_3S_8$ (M – Mn, Fe, Co, Ni) thiospinels with low thermal conductivity

Oleksandr Cherniushok[1], Oleksandr V. Smitiukh[2], Janusz Tobola[3], Rafal Knura[1], Oleg V. Marchuk[2], Taras Parashchuk[1*], Krzysztof T. Wojciechowski[1*]

[1]Thermoelectric Research Laboratory, Department of Inorganic Chemistry, Faculty of Materials Science and Ceramics, AGH University of Science and Technology, Mickiewicza Ave. 30, 30-059 Krakow, Poland
[2]Department of Chemistry and Technology, Volyn National University, Voli Ave 13, Lutsk, 43025, Ukraine
[3]Faculty of Physics and Applied Computer Science, AGH University of Science and Technology, Mickiewicza Ave. 30, 30-059 Krakow, Poland

*Corresponding author(s):

Taras Parashchuk, E-mail: parashchuk@agh.edu.pl.

Krzystof T. Wojciechowski, E-mail: wojciech@agh.edu.pl

Table of contents

Figure S1. Powder XRD patterns of $Cu_2MHf_3S_8$ samples after synthesis and pellets after measurements.

Figure S2. Heating and cooling data of electrical conductivity (a), Seebeck coefficient (b), and thermoelectric power factor *PF* (c) for $Cu_2MHf_3S_8$.

Figure S3. The optical absorption spectra versus photon energy of $Cu_2MHf_3S_8$ (M=Mn, Fe, Co, and Ni) thiospinels at room temperature.

Equations S1-S8. Details of elastic properties calculations.

Equations S9-S10. Details of thermal transport properties calculations.

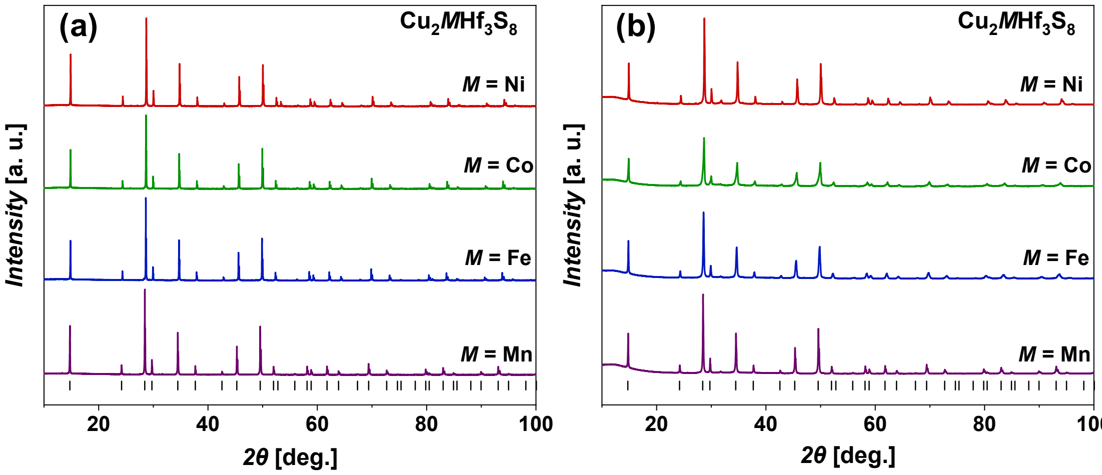

Figure S1. Powder XRD patterns of $Cu_2MHf_3S_8$ samples after synthesis (a) and pellets after measurements (b).

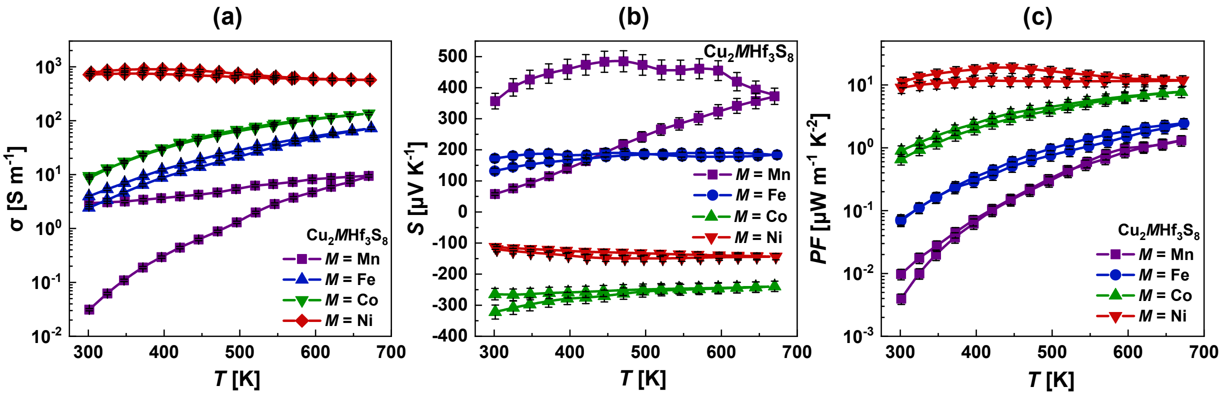

Figure S2. Heating and cooling data of electrical conductivity (a), Seebeck coefficient (b), and thermoelectric power factor *PF* (c) for $Cu_2MHf_3S_8$.

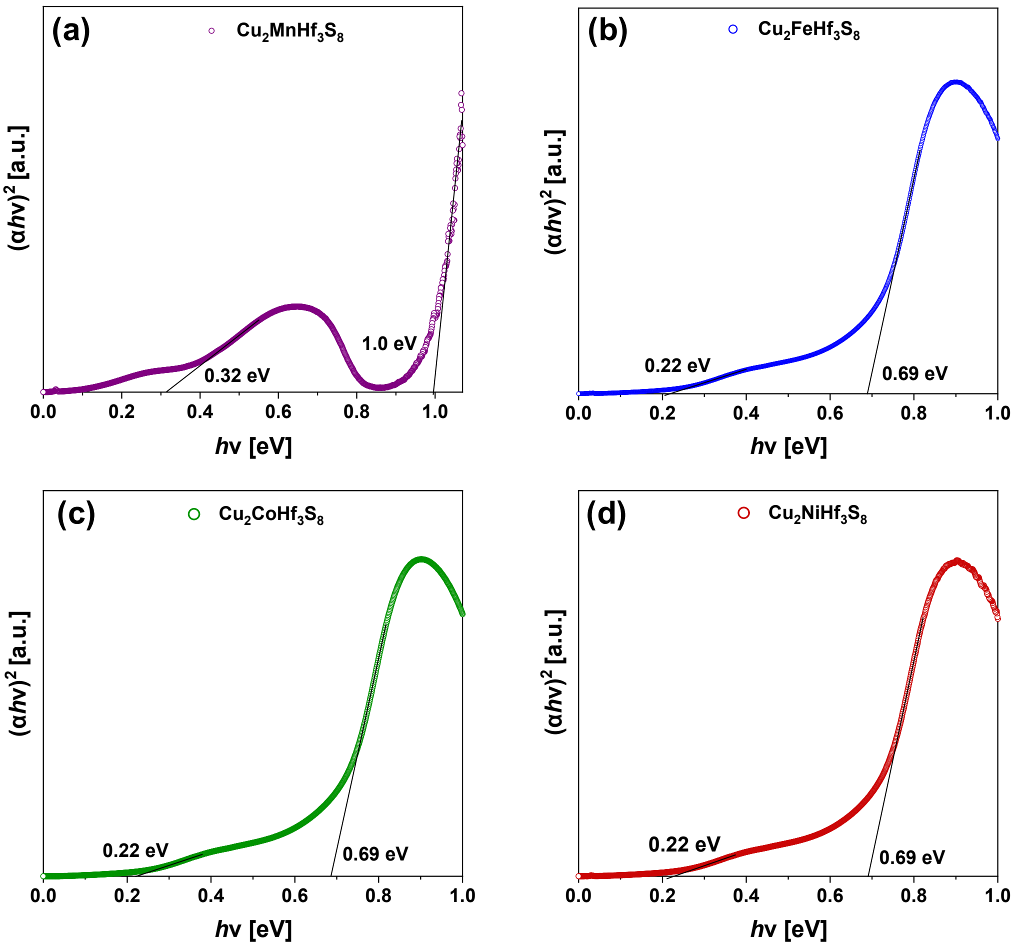

Figure S3. The optical absorption spectra versus photon energy of $Cu_2\mathit{M}Hf_3S_8$ (*M*=Mn, Fe, Co, and Ni) thiospinels at room temperature.

Elastic properties

The bulk modulus was calculated using the following equation [1-3]:

$$B = \rho\left(\upsilon_L^2 - \frac{4}{3}\upsilon_T^2\right). \quad \text{(S1)}$$

where ρ is the material density.

The shear modulus was calculated as:

$$G = \upsilon_T^2 \rho. \quad \text{(S2)}$$

The Young's modulus is calculated as:

$$E = \frac{9BG}{3B + G}. \tag{S3}$$

The Poisson's ratio is calculated as:

$$\nu = \frac{E - 2G}{2G}. \tag{S4}$$

The Debye temperatures were calculated using the following expression [4]:

$$\Theta_D = \frac{h}{k_B}\left[\frac{3n}{4\pi}\left(\frac{N_A \rho}{M}\right)\right]^{1/3} v_m, \tag{S5}$$

where h is Planck's constant, k_B is Boltzmann's constant, N_A is Avogadro's number, M is the molecular weight, n is the number of atoms in the molecule, and v_m is the averaged wave velocity integrated over several crystal directions [5]:

$$v_m = \left[\frac{1}{3}\left(\frac{2}{v_t^3} + \frac{1}{v_l^3}\right)\right]^{-1/3}, \tag{S6}$$

where v_l and v_t are the longitudinal and transverse sound velocities.

Effect of the difference between the longitudinal and transverse speed of sound on the Grüneisen parameter

Grüneisen parameters γ were calculated using the following equation [5]:

$$\gamma = \frac{3}{2}\left(\frac{1+\nu}{2-3\nu}\right). \tag{S7}$$

where ν is the Poisson ratio. The square of the ratio of the longitudinal and transverse speed of sound $\left(\frac{v_l}{v_t}\right)^2$ can be found using the following equation [5]:

$$\left(\frac{v_l}{v_t}\right)^2 = \left(\frac{2-2\nu}{1-2\nu}\right) \tag{S8}$$

Let us consider that $\alpha = \left(\frac{v_l}{v_t}\right)^2$. Then α is greater if the ratio v_L:v_S is greater. After solving the system of equations (S7) and (S8), we obtain: $\gamma = \frac{\frac{3}{2}+\frac{\frac{3}{2}(2-\alpha)}{2-2\alpha}}{2-\frac{3(2-\alpha)}{2-2\alpha}}$. After plotting the derivative $\frac{d\gamma}{d\alpha}$ and analyzing this dependence we have found that it is an increasing function over the interval (1; 10) (α < 1 is not considered because it would mean that v_L<v_T, the values of α > 10 were not considered as $v_L > \sqrt{10}\, v_T$ is very unlikely). The performed analysis indicates that γ increases with increasing α, therefore γ increases with increasing $\frac{v_l}{v_t}$ ratio.

Thermal transport properties

From the kinetic theory, the lattice thermal conductivity is expressed as [6]:

$$\kappa_{lat} = \frac{1}{3} C_V v_m l_{ph} \tag{S9}$$

where κ_{lat} is experimental lattice thermal conductivity, C_V is the specific heat at constant volume, v_m is the average sound velocity. Considering this, the phonon mean free path can be calculated using the following equation [7]:

$$l_{ph} = \frac{3\kappa_{lat}}{C_V v_m} \tag{S10}$$

For the calculation of l_{ph} at 298 K, the κ_{lat} was assumed equal to the measured κ due to very low electrical conductivity (σ < 10 S/cm), specific heat capacity was estimated using the Dulong-Petit approximation, and average sound velocity was obtained from the acoustic data of longitudinal v_l and transverse v_t sound velocities using Equation S9.

References

[1] Y.I. Ravich, B.A. Efimova, I.A. Smirnov, Semiconducting Lead Chalcogenides, Springer US, 1970. https://doi.org/10.1007/978-1-4684-8607-0.

[2] B.M. Askerov, Electron Transport Phenomena in Semiconductors, WORLD SCIENTIFIC, 1994. https://doi.org/10.1142/1926.

[3] Thermoelectrics Handbook: Macro to Nano - 1st Edition - D.M. Rowe - H, (n.d.).

https://www.routledge.com/Thermoelectrics-Handbook-Macro-to-Nano/Rowe/p/book/9780849322648 (accessed February 13, 2021).

[4] O.L. Anderson, A simplified method for calculating the debye temperature from elastic constants, J. Phys. Chem. Solids. 24 (1963) 909–917. https://doi.org/10.1016/0022-3697(63)90067-2.

[5] D.S. Sanditov, V.N. Belomestnykh, Relation between the parameters of the elasticity theory and averaged bulk modulus of solids, Tech. Phys. 56 (2011) 1619–1623. https://doi.org/10.1134/S106378421111020X.

[6] T. M. Tritt, *Thermal conductivity: theory, properties, and applications*, Springer Science & Business Media, 2005.

[7] H. Xie, S. Hao, S. Cai, T.P. Bailey, C. Uher, C. Wolverton, V.P. Dravid, M.G. Kanatzidis, Ultralow thermal conductivity in diamondoid lattices: high thermoelectric performance in chalcopyrite $Cu_{0.8+y}Ag_{0.2}In_{1-y}Te_2$, Energy Environ. Sci. 13 (2020) 3693–3705. https://doi.org/10.1039/D0EE02323J.
